# Conflict of Interest in Spine Research Reporting

**DOI:** 10.1371/journal.pone.0044327

**Published:** 2012-08-31

**Authors:** Brian P. Walcott, Sameer A. Sheth, Brian V. Nahed, Jean-Valery Coumans

**Affiliations:** Department of Neurosurgery, Massachusetts General Hospital and Harvard Medical School, Boston, Massachusetts, United States of America; University of Michigan, United States of America

## Abstract

**Background:**

Medical studies are more likely to report favorable findings when a conflict of interest is declared. We aim to quantify and determine the effect of author disclosure of conflict of interest on scientific reporting.

**Methods:**

Abstracts from an international spine research meeting (North American Spine Society 2010) were selected that specifically evaluated a device, biologic, or proprietary procedure. They were then made anonymous to reviewers. An item of interest was established in each of the abstracts in order to standardize evaluation. Next, three blinded reviewers independently rated the abstracts as favorable, neutral, or unfavorable with regard to the item of interest. Additionally, the blinded reviewers attempted to predict whether a related disclosure was made. The meeting disclosure index was used to tabulate the minimum US dollar value attributable to disclosures.

**Results:**

Of the 344 total abstracts, 76 met inclusion criteria. In 79%, a related conflict of interest was reported. The amount of the disclosure was incompletely reported in 30% of cases. Where available, it averaged a cumulative minimum of $219,634 USD per abstract. The results of the abstracts were judged to be favorable, neutral, and unfavorable in 63%, 32% and 5% of abstracts, respectively. There was no correlation between the presence of a related disclosure and the findings of the studies (p = 0.81), although interpretation of this is limited by a small sample size and an overall apparent bias to report favorable studies. Additionally, the blinded reviewers were unable to predict whether a related disclosure was made (p = 0.40).

**Conclusion:**

No association existed between the presence of a related disclosure and the results of the studies. While the actual compliance with reporting a potential conflict of interest is unable to be determined, the value amount related to the disclosures made was inadequately reported according to meeting guidelines.

## Introduction

Conflict of interest, as it pertains to medicine, occurs when a physician interest compromises the integrity of the physician-patient relationship. There is a societal and patient centered expectation that the treating provider is free from these conflicts when rendering decisions about medical care. Paradoxically, there is also the realization that in the current healthcare delivery system, potential conflicts are unavoidable for the most part. Indeed, there are many beneficial aspects of commercial alliance, including research support, clinical product support, and innovation. Therefore, public transparency is expected to exist in order to improve the impartiality of a *de facto* impartial arrangement. New legislation, such as the Physician Payments Sunshine Act, has recently been enacted to improve transparency of these physician-industry relationships through mandatory public disclosure. [1]

For the spine surgeon in particular, affiliation with industry raises concerns for impartiality and conflict of interest. Medical devices and biologics are rapidly growing staples in the practices of many spine surgeons and have significant costs at both the individual and national level. For example, while bone morphogenic protein (BMP) is currently only FDA-approved for use in the lumbar spine, its nationwide usage has increased from 0.69% of all-level fusions in 2002 to 24.89% of all-level fusions in 2006. [2] Its usage has been implicated in complications in the anterior cervical region, while also being associated with greater hospital charges. [2], [3] The rates of spinal fusion overall have also increased dramatically over recent years and are inherently more costly procedures than decompression procedures alone. [4]–[6] The increased rate of spinal fusion surgery has been linked to an increased risk of major complications, mortality, and resource use in older populations. [5] With poor outcome measures and a paucity of data to suggest improved outcomes associated with the increased use of these devices/biologics, interest in the potential for conflicts of interest to contribute to this phenomenon has grown. [7]

Recognizing this, several parent organizations and governing bodies have imposed rigorous policies to increase transparency mandating full disclosure with any research publications, research projects, meetings, and educational programs. Some of these policies even require quantitative, categorical identification of financial relationships. [8] In spite of this requirement, there is significant variability in the reporting of financial conflicts of interest at national and international meetings presenting spine research, making interpretation difficult. [9]

Beyond patient care and healthcare economics, financial relationships may influence the outcome of scientific reporting. Studies have demonstrated that investigators with financials relationships with the manufacturers of a drug or device are significantly more likely to report “positive” evaluations of the intervention as compared to being “neutral” or “negative”. [10]–[12] Relationships with industry have been associated with restrictions on publication and data sharing in the scientific community. [13] Within spine research, in particular, industry funded studies have been associated with a statistically greater likelihood of positive results than studies with other funding sources. [14], [15] While these relationships with industry have been described, along with compliance to reporting the same, no quantitative analysis has yet been performed. Herein, we analyze research abstracts from a large, international spine research meeting known for its rigorous disclosure requirements to identify the influence of potential conflicts of interest.

## Materials and Methods

Each oral presentation abstract from the 2010 North American Spine Society (North America’s largest multidisciplinary spine society) annual meeting was reviewed via an online, web-based archive. An independent individual, blinded to the subsequent abstract evaluation, selected all abstracts that specifically evaluated a device, biologic, or proprietary procedure. Identifiable information regarding author identity, institution and geographical location were removed resulting in anonymous abstracts formatted with title and the body of text. An item of interest was established in each of the abstracts to standardize evaluation. (For example, if bone morphogenic protein was identified as an item of interest, then it would represent the fulcrum point to later assess outcomes).

Next, three blinded reviewers independently rated the abstracts as favorable, neutral, or unfavorable with regard to the item of interest. The mode was used to generate a categorical variable. Additionally, the blinded reviewers attempted to predict whether a related disclosure was made.

Finally, the 2010 NASS annual meeting disclosure index (release date May 10, 2010) was used to tabulate the minimum US dollar value attributable to related royalties, consulting, speaking & travel arrangements, scientific advisory board work, and grants or research support, creating a matrix with the abstract authorship information. [16] Levels of financial association were previously categorized by the NASS as (A) $100 to 1000, (B) $1001 to 10,000, (C) $10,001 to 25,000, (D) $25,001 to 50,000, (E) $50,001 to 100,000, (F) $100,001 to 500,000, (G) $500,001 to 1,000,000, (H) $1,000,001 to 2,500,000, and (I) greater than $2,500,000. Each abstract’s minimum cumulative association was determined by summating the authors’ individual values based on the minimum value in the category range. In the absence of exact dollar amounts, the actual financial association lies somewhere between the minimum and maximum dollar amounts for each category. Private investments were noted but not included in the analysis if they were not obtained as an obvious direct benefit of the relationship. Non-commercial grants were excluded (e.g. originating from the National Institutes of Health). Commercial employee status was identified as an obvious conflict of interest. Relationships outside of a one-year time period were excluded. Statistical analysis of the distribution of results was performed with Prism 5 for Mac (GraphPad Software, Inc). Pearson’s chi-squared test was utilized to analyze observer determination of favorability and the occurrence of study disclosure. Fisher’s exact test (two-sided) was used to analyze observer prediction of the occurrence of study disclosure and actual occurrence. Fleiss’ kappa was utilized to assess the degree of inter-observer agreement. Significance was pre-defined at p<0.05.

## Results

**Figure 1:**
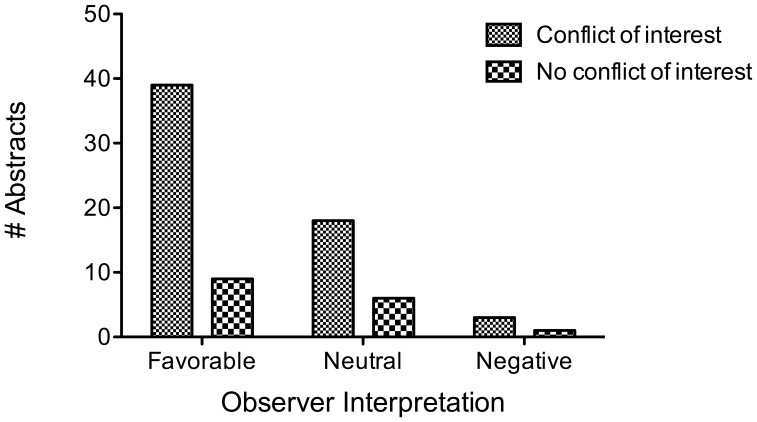
Observer interpretation of study favorability. Three independent and blinded researchers assessed the favorability of the results of an abstract with respect to a designated item of interest. There was no association with these ratings and whether a related conflict of interest was present (p = 0.81).

**Figure 2:**
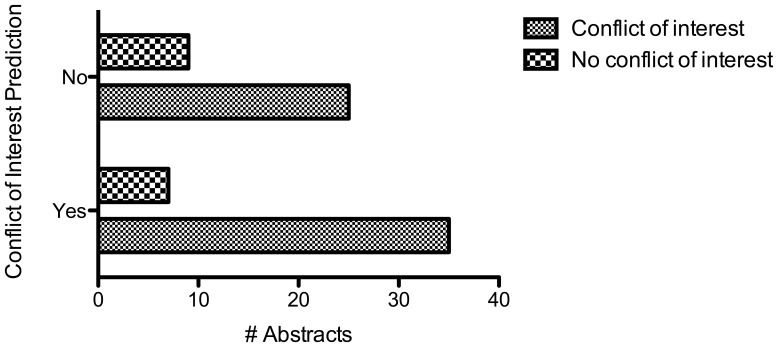
Observer prediction of disclosure declaration. Three independent and blinded researchers were unable to predict whether a related conflict of interest was present for each abstract (p = 0.40).

There were 344 abstracts in total accepted for presentation. Of these, 76 met inclusion criteria. In 79% of these abstracts, a related conflict of interest was reported. The amount of disclosure was incompletely reported in 30% of cases. The reason for incomplete disclosure was not reporting the actual dollar amount category of the disclosure (required by meeting guidelines). Where available, it averaged a cumulative minimum of $219,634 USD per abstract. The results of the abstracts were judged by the reviewers to be favorable, neutral, and unfavorable in 63%, 32% and 5% of abstracts (mode), respectively. Inter-observer agreement was good among the three observers (Fleiss’ kappa, 0.411). There was no correlation between the presence of a conflict of interest and the findings of the studies (p = 0.81). [Figure 1] Additionally, the blinded reviewers were unable to predict whether a conflict of interest existed (p = 0.40). [Figure 2]

Several notable levels of disclosure were identified: authors were identified as receiving royalty payments greater than $500,000 (n = 5), 1,000,000 (n = 2), and 2,500,000 (n = 2). One author was identified as reporting disclosures related to trips or travel of greater than $500,000.

## Discussion

Relationships with device and biologic companies have and will continue to exist in medicine; the purpose of this study was to assess the implications of these relationships on the presentation of scientific research in the spine community. While much has been written on the potential influence of conflict of interest on medical and scientific work, our study is the first to use quantitative analysis to analyze actual dollar amount estimates of related financial conflicts of interest (self reported). Our analysis failed to find a correlation between the perceived findings of the abstract and the presence of a potential conflict of interest. Additionally, because the choice of words and the tone of the abstract can lead to a perception of bias, we attempted to increase the reliability of our findings by asking each independent, blinded reviewer to guess whether a conflict of interest existed based on the tone of the abstract. In this study, we report that despite a high percentage of disclosed conflicts of interest for research studies summating to a significant amount of money, no apparent influence on reporting of their outcome was found.

Physicians and scientists are held to the highest ethical standard of maintaining independence in decision making and data analysis. Speaking honoraria, consulting contracts, and royalty payments, must never distort clinical care and research integrity. Studies have demonstrated that conflict of interest may have conscious or unconscious effects that lead to the promotion of devices or off-label use of devices. As a result there is a strong perception that medical decisions and the completeness and accuracy of scientific study design and data may be marred by the accountability to outside commercial interests. Our study supports the hypothesis that independence in medical decision making may be preserved despite related conflicts of interest. Critics may argue that independence is preserved only to a certain monetary level, yet the available data does not permit an accurate dose-response relationship analysis.

Several limitations to this study should be noted. Most apparent is that it is the analysis of a single year and single scientific meeting. However, precedence for this type of focused analysis has been established. [17] Comparison analysis between multiple meetings of the same year have identified discrepancies in conflict of interest reporting, however not all meetings in a given year require quantification of the disclosure as was necessary for our analysis. [9]

Another inherent limitation of this study was the fact that only accepted abstracts were analyzed. It is well known that scientific studies with positive findings are published more often, and more quickly, than trials with negative findings, irrespective of their validity. [18], [19] Also known at the “winner’s curse”, the current system of biomedical literature evaluation, publication, and dissemination distorts the reality of scientific research via a very strong publication bias in favor of positive results. [20], [21] With a bias towards positive studies, it is difficult to assess the influence of conflict of interest on study outcome when the majority of studies presented in an overall small sample size are favorable. Secondary to this power limitation, a type II error cannot be excluded.

Even if no commercial conflicts of interest are reported, there may be financial incentives that influence medical practice that are not currently required to be reported by any scientific organization. That is, differential reimbursement patterns exist for surgical procedures that are difficult to explicitly account for, as universal fee and charge reporting is not required. Generally, it is well recognized that spinal fusion procedures reimburse more favorably than do non-fusion spinal surgery. In the very select Medicare population studied by Deyo et al., reimbursement for a non-fusion spinal decompression was estimated at $600–800 USD. [5] For complex fusions, the reimbursement can be up to 10-fold greater. [5], [22]

Finally, disclosures are not always made, even when significant conflicts of interest exist. [9], [17] There exist no universal database to allow for centralized reporting and no systems exist to validate the claims of physicians.

### Conclusion

For the 2010 NASS annual meeting abstracts that evaluated a device, biologic, or proprietary procedure, no association existed between the presence of a conflict of interest and the results of the studies. While the actual compliance with reporting a conflict of interest is undetermined, the value amount related to the disclosures made was inadequately reported.
